# Allyl Isothiocyanate Suppresses the Proliferation in Oral Squamous Cell Carcinoma via Mediating the KDM8/CCNA1 Axis

**DOI:** 10.3390/biomedicines11102669

**Published:** 2023-09-29

**Authors:** Cheng-Chih Hsieh, Cheng-Yu Yang, Bo Peng, Sien-Lin Ho, Chang-Huei Tsao, Chih-Kung Lin, Chun-Shu Lin, Gu-Jiun Lin, Heng-Yi Lin, Hung-Chi Huang, Szu-Chien Chang, Huey-Kang Sytwu, Wei-Tso Chia, Yuan-Wu Chen

**Affiliations:** 1 Department of Pharmacy, Kaohsiung Veterans General Hospital, Kaohsiung 813, Taiwan; 2 School of Pharmacy and Institute of Pharmacy, National Defense Medical Center, Taipei 114, Taiwan; 3 School of Dentistry, National Defense Medical Center, Taipei 114, Taiwan; 4 Department of Oral and Maxillofacial Surgery, Tri-Service General Hospital, Taipei 114, Taiwan; 5 Department of Microbiology and Immunology, National Defense Medical Center, Taipei 114, Taiwan; 6 Department of Medical Research, Tri-Service General Hospital, Taipei 114, Taiwan; 7 Division of Anatomic Pathology, Taipei Tzu Chi Hospital, New Taipei City 231, Taiwan; 8 Department of Radiation Oncology, Tri-Service General Hospital, National Defense Medical Centre, Taipei 114, Taiwan; 9 Graduate Institute of Clinical Medicine, College of Medicine, Taipei Medical University, Taipei 110, Taiwan; 10 Department of Biology and Anatomy, National Defense Medical Center, Taipei 114, Taiwan; 11 Department of Dentistry, Cardinal Tien Hospital, New Taipei City 231, Taiwan; 12 Department of Dentistry, Hualien Armed Forces General Hospital, Hualien 971, Taiwan; 13 Department of Dentistry, Kaohsiung Armed Forces General Hospital, Kaohsiung 813, Taiwan; 14 National Institute of Infectious Diseases and Vaccinology, National Health Research Institutes, Zhunan, Miaoli 350, Taiwan; 15 Department of Orthopedics, National Taiwan University Hospital Hsin-Chu Branch, Hsinchu 302, Taiwan; 16 Department of Nursing, Yuan Pie University of Medical Technology, Hsinchu 302, Taiwan; 17 Tri-Service General Hospital, Taipei 114, Taiwan

**Keywords:** oral cancer, allyl isothiocyanate, patient-derived tumor xenograft (PDTX), KDM8/JMJD5, H3K36me2, CCNA1

## Abstract

The dysregulated expression of cyclin genes can lead to the uncontrolled proliferation of cancer cells. Histone demethylase Jumonji-C domain-containing protein 5 (KDM8, JMJD5) and cyclin A1 (CCNA1) are pivotal in cell cycle progression. A promising candidate for augmenting cancer treatment is Allyl isothiocyanate (AITC), a natural dietary chemotherapeutic and epigenetic modulator. This study aimed to investigate AITC’s impact on the KDM8/CCNA1 axis to elucidate its role in oral squamous cell carcinoma (OSCC) tumorigenesis. The expression of KDM8 and CCNA1 was assessed using a tissue microarray (TMA) immunohistochemistry (IHC) assay. In vitro experiments with OSCC cell lines and in vivo experiments with patient-derived tumor xenograft (PDTX) and SAS subcutaneous xenograft tumor models were conducted to explore AITC’s effects on their expression and cell proliferation. The results showed elevated KDM8 and CCNA1 levels in the OSCC patient samples. AITC exhibited inhibitory effects on OSCC tumor growth in vitro and in vivo. Additionally, AITC downregulated KDM8 and CCNA1 expression while inducing histone H3K36me2 expression in oral cancer cells. These findings underscore AITC’s remarkable anticancer properties against oral cancer, highlighting its potential as a therapeutic option for oral cancer treatment by disrupting the cell cycle by targeting the KDM8/CCNA1 axis.

## 1. Introduction

Oral squamous cell carcinoma (OSCC) is a type of cancer affecting the head and neck, which has a rising trend of incidence in the world [1,2]. The development of OSCC is frequently linked to various risk factors, including but not limited to smoking, alcohol consumption, chewing habits, and infection with high-risk human papillomavirus [1,2]. Despite the considerable benefits observed in surgical procedures, radiation therapy, and chemotherapy, the 5-year survival rate for patients with OSCC remains below approximately 50% [3]. As a result, the identification of new prognostic factors and treatments is of utmost importance to impede the progression of OSCC. Due to their safety, natural compounds have recently become a focal point in the quest for anticancer drugs as potential alternatives [4]. Previous studies have indicated that CCNA1 is a cyclin-dependent protein kinase known as a serine/threonine kinase, predominantly found in the testis. It belongs to the highly conserved cyclin family, which shows periodic fluctuations in abundance during the cell cycle. In the eukaryotic cell cycle, the CCNA1 protein acts as a regulatory subunit for cyclin-dependent kinases (CDKs). CCNA1 activates CDK2 by binding specifically to it, leading to the phosphorylation of several target proteins that facilitate the progression through the G1/S and G2/M phases of the cell cycle [5,6,7]. CCNA1 plays a crucial role in regulating cell cycle progression, and its suppression in leukemia cells has been shown to impede cell growth [6]. The overexpression of CCNA1 has been linked to unfavorable prognoses in various cancers, including bladder urothelial carcinomas [8], esophageal squamous cell carcinoma [9], as well as head and neck cancer [10,11,12].

The aberration of chromatin modifications of histone tails leads to carcinogenesis [13]. Jumonji C domains protein families have been identified as major contributors to various human cancers via epigenetic remodeling [14,15]. Jumonji-C domain-containing protein 5 (JMJD5), renamed KDM8, is involved in embryonic development [16], the metabolic regulator [17], osteoclastogenesis [18], circadian rhythm regulation [19], and tumorigenesis [20]. KDM8 functions as a histone demethylase that specifically removes methyl groups from lysine 36 on histone H3 (H3K36), resulting in the modulation of gene expression. KDM8 is highly expressed in various types of cancer such as breast, lung, stomach, prostate, colon, and oral cancers [20,21,22,23,24,25,26,27]. The upregulation of KDM8 in these cancers has been linked with enhanced cell proliferation, migration, and invasion, which indicates its involvement in tumor progression. Furthermore, a research study found that the inhibition of KDM8 can impede metastasis and prompt apoptosis in oral squamous cell carcinoma through the regulation of the p53/NF-κB pathway [20]. In our previous report, we demonstrated a correlation between the high expression of KDM8 and an unfavorable prognosis in OSCC, and the downregulation of JMJD5 could inhibit proliferation in the oral cancer preclinic model [27]. Moreover, KDM8 has been revealed to positively regulate CCNA1 in cancer cell proliferation [13]. KDM8 and CCNA1 may have a function in the regulation of the p53 pathway and affect cell cycle progression and DNA damage responses by interacting with p53. Considering these discoveries, both KDM8 and CCNA1 are potential targets for cancer treatment.

Previous research has shown that natural compounds derived from plants possess chemopreventive, anticancer, and antimetastatic properties and functions [28]. Isothiocyanates (ITCs) are well-established and have been reported to exhibit anticancer effects in human cancers [29,30,31]. These compounds are present in a variety of cruciferous vegetables, including cauliflower, brussels sprouts, kale, cabbage, horseradish, and wasabi [31,32]. Allyl isothiocyanate (AITC; 3-isothiocyanato-1-propene, CH2CHCH2NCS) is responsible for the pungent flavor of mustard, horseradish, radish, and wasabi. AITC, which is a sulfur-containing organic compound, is a product of the enzymatic hydrolysis of the glucosinolate sinigrin. Research has shown that AITC can hinder cancer cell progression by impeding cell growth, proliferation, migration, and invasion [31,32]. AITC has been found to regulate DNA methylation, a process that involves the addition of a methyl group to the DNA molecule and can reduce DNA methylation in cancer cells. This leads to the reactivation of tumor suppressor genes and inhibition of cancer cell growth [33,34,35,36,37]. AITC has also been found to inhibit the activity of histone deacetylases (HDACs), which are the enzymes involved in regulating histone modification [34,38,39]. By inhibiting HDAC activity, AITC can increase histone acetylation, alter gene expression, and prevent cancer cell growth [34]. Furthermore, AITC has been shown to induce apoptosis and G2/M phase arrest in human brain malignant glioma GBM 8401 cells [40] as well as apoptotic death in human cisplatin-resistant oral cancer cells [29]. Various preclinical studies have shown that AITC can inhibit lung, breast, gastric, prostate, and bladder cancer, but this does not reflect in the pilot trials performed in humans to date [41,42,43].

The lack of sufficient tumor models that have clinical relevance poses a significant obstacle to the development of effective therapies for oral cancer. It is crucial to establish PDTX models that accurately represent the phenotypic and genotypic characteristics of oral cancer. PDTX, an innovative preclinical animal model, consistently maintains the tumor’s morphology and genetic stability and is one of the most promising platforms for simulating human cancer and its complexity. The histopathology of PDTX tumors is very similar to the histopathology of donor lesions. A large amount of evidence, including mutation status, transcriptome, histology, polymorphism, and copy number variation with high fidelity, also supports the view that the PDTX model is very similar to human tumors’ pathophysiology than the traditional cancer-derived xenograft model [44,45].

AITC has been shown to inhibit several common cancer types both in vitro and in vivo [46,47,48], but there are limited studies on its inhibitory effects on oral cancer cells [29,49]. As a result, this study utilizes a preclinical PDTX model and an oral cancer cell line to explore whether AITC’s anticancer effects on cell proliferation in oral cancer are modulated through KDM8 and CCNA1.

## 2. Materials and Methods

### 2.1. Chemicals and Reagents

The following chemicals and reagents were procured from Sigma-Aldrich (St. Louis, MO, USA): AITC, methylene blue, propidium iodide (PI), ethanol, DMSO, and isopropanol. A concentration of 10 mM AITC was prepared by dissolving it in DMSO and storing it at 4 °C. RPMI 1640 and fetal bovine serum (FBS) were obtained from Biological Industries (Beit-Haemek, Israel), while RIPA buffer was purchased from Millipore (Burlington, MA, USA). The present study utilized primary antibodies against KDM8 (AVIVA SYSTEMS BIOLOGY; ARP58120_P050), H3K36me2 (GeneTex; GTX54108), Cyclin A1 (GeneTex; GTX02524), and GAPDH (GeneTex; GTX100118), as well as secondary antibodies (GeneTex; GTX213110-01, GTX213111-01).

### 2.2. Tumor Specimens

Samples of tumors and adjacent normal oral mucosa were collected from 27 patients with OSCC at the Tri-Service General Hospital of National Defense Medical Center (Taipei, Taiwan) with their consent and in compliance with Institutional Review Board protocols (TSGH-2-105-05-004). Tumor tissue samples were obtained from archived formalin-fixed, paraffin-embedded (FFPE) specimens collected during diagnosis and stored at hospital pathology departments. The tissue microarray (TMA) procedure was carried out using a method previously described [27].

### 2.3. Immunohistochemistry

The immunohistochemical staining protocol was utilized to evaluate KDM8 antibody binding in all tissue specimens, and the resulting staining scores were determined according to previously published methods [27]. Specifically, the immunostaining score was calculated by multiplying the score for stained tumor cells by the intensity score. The intensity of immune reactivity towards tumor cells was graded on a 0–3 scale, with 0 indicating no staining, 1 indicating weak intensity, 2 indicating moderate intensity, and 3 indicating strong intensity. For each intensity score, the percentage of tumor cells exhibiting nuclear or cytosolic staining was graded on a 5-point scale, with 0 indicating no staining and 4 indicating staining in 75–100% of cells.

### 2.4. Cell Lines and Cell Culture

The cell lines were cultured in RPMI 1640 media supplemented with 10% fetal bovine serum, 1% penicillin/streptomycin, and 2 mmol/L L-glutamine, following established protocols [27]. The SCC25 tongue cancer cell line was obtained from the American Type Culture Collection (ATCC), while the SAS tongue cancer cell line was kindly provided by Dr. Lo from the Institute of Oral Biology, Department of Dentistry, National Yang-Ming University, Taipei, Taiwan.

### 2.5. In Vitro Cell Proliferation Assay

The antitumor effect of AITC on oral cancer cell growth was performed by the methylene blue dye assay, as described previously [50].

### 2.6. Cell Cycle Analysis

The study utilized a cell cycle assay method previously described in the literature [51]. Briefly, SAS oral cancer cells were seeded in tissue culture plates at a density of 1 × 10^6^ cells/dish and subjected to flow cytometry analysis using the FACSCalibur instrument (Becton Dickinson, Franklin Lakes, NJ, USA). The cells were treated with different concentrations of AITC (0, 25, and 50 μM) for 24 and 48 h. Subsequently, the cells were harvested, fixed in chilled ethanol overnight at 4 °C, washed with PBS, and resuspended in PBS. The cells were then incubated with 0.5 mL of PI/RNase for 15 min at room temperature.

### 2.7. Western Blot Analysis

A previously published protocol [50] was followed to perform Western blot analysis. In summary, cell pellets were lysed directly in RIPA buffer with added protease and phosphatase inhibitors (Calbiochem, San Diego, CA, USA). The protein concentration of the resulting supernatants was measured using a BCA protein assay kit (Thermo Scientific, Waltham, MA, USA). Afterward, 30 μg of cell lysate protein was loaded onto each lane of a 10% SDS-PAGE gel, separated, and then transferred onto a polyvinyldifluoride membrane (Amersham, Freiburg im Breisgau, Germany). Finally, specific antibodies were used to probe the membranes for target proteins.

### 2.8. Establishment of PDTX Models and Treatment Protocol

The methods used to establish PDTX were outlined in a previous study [51]. In brief, tumor fragments were repeatedly washed in penicillin/streptomycin culture medium. All surgical instruments were sterilized before use. The procedure involved subcutaneous transplantation by the operating personnel, also known as a survival surgery. The surgical process included wiping the Biose-cure operating table and all tools with Virkon and injecting 100 μL of ketamine mixed with Rompun into the abdominal cavity. Subcutaneous transplantation was performed after anesthesia took effect. After implantation, the wound was sutured with stitches. Postoperatively, the wound site was continuously monitored until the experiment’s completion (see Figure S1 for the workflow), and the histological patterns of the tumor tissue were verified using H&E staining. The establishment of PDTX models and treatment protocols was approved by ethical guidelines and the Institutional Review Board (TSGH-2-102-05-111). Tumor specimens were obtained from patients with OSCC during their initial surgical treatment and were classified as T4aN2b based on World Health Organization criteria. These tumors were then maintained in RPMI 1640 medium and implanted subcutaneously into nonobese diabetic/severe combined immunodeficiency/gamma (NSG) mice aged 6–10 weeks. The xenograft models were monitored at least twice a week, and the tumor volume was calculated using the formula V = 1/2 × (length × width^2^). Tumor tissues were removed and transplanted serially if the tumor volume reached approximately 3000 mm^3^. When the tumor volume reached approximately 500 mm^3^, mice with seventh-generation SC179-PDTXs were randomly assigned to three groups: one receiving AITC (50 mg/Kg/daily, n = 4), one receiving cisplatin (10 mg/Kg/daily, n = 4), and a third group receiving Phosphate buffered saline (PBS, n = 4) as a control. Treatment was administered by intraperitoneal injection for 24 days, and body weight and tumor volume were measured at least twice a week. At the end of the treatment, the mice were sacrificed, and tumors were removed, weighed, and observed. The animal experiments were approved by the National Defense Medical Center Institutional Animal Care and Use Committee, Taipei, Taiwan (IACUC; 16-244 and 18-027).

### 2.9. Mouse SAS Xenograft Model

Previously described methods [50] were used to perform SAS xenograft animal models. Approval for all experiments was obtained from the National Defense Medical Center Institutional Animal Care and Use Committee, Taipei, Taiwan (IACUC; 16-244). Nonobese diabetic/severe combined immunodeficiency (NOD/SCID) mice that were eight weeks old were housed in microisolators under specific pathogen-free conditions. The mice were divided into three groups: the AITC treatment group (n = 4), which received 50 mg/Kg body weight/daily intraperitoneal (i.p.) treatment; the positive control group (n = 4), which received 15 mg/Kg body weight/daily i.p. treatment of 5-Flurouracil (5-FU); and the vehicle control group (n = 4), which received PBS treatment. Each group of mice was subcutaneously injected with 2 × 10^6^ SAS oral cancer cells. Drug treatments began on the third day after the tumor injection and continued until day 21. Tumor size was monitored at least twice a week, and tumor volume was calculated using the formula V = 1/2 × (length × width^2^). At the end of the experiment, the mice were sacrificed, and the tumors were excised, weighed, and examined.

### 2.10. UALCAN

UALCAN (http://ualcan.path.uab.edu/index.html, URL (accessed on 17 July 2023)) is a web-based tool that provides a thorough examination of gene expression data derived from The Cancer Genome Atlas (TCGA) database [52,53]. The tool’s “TCGA Gene analysis” module was employed to investigate the mRNA levels of KDM8/JMJD5 in head and neck squamous carcinoma (HNSC) patients and healthy individuals. The analysis also explored the correlation of these levels with clinicopathological parameters. The TCGA HNSC dataset, comprising genetic data from 520 individuals, was used for the analysis. The significance of the findings was determined at *p* < 0.05.

### 2.11. Statistical Analysis

The statistical analysis of the data was carried out using the GraphPad Prism software (GraphPad Software 8.0, San Diego, CA, USA). The statistical significance of the results was determined by performing unpaired, two-tailed Student’s *t*-tests. *p* < 0.05 was considered statistically significant.

## 3. Results

### 3.1. KDM8 and CCNA1 Expression Exhibited a Significant Increase in Both Human OSCC and Cell Lines

**Figure 1:**
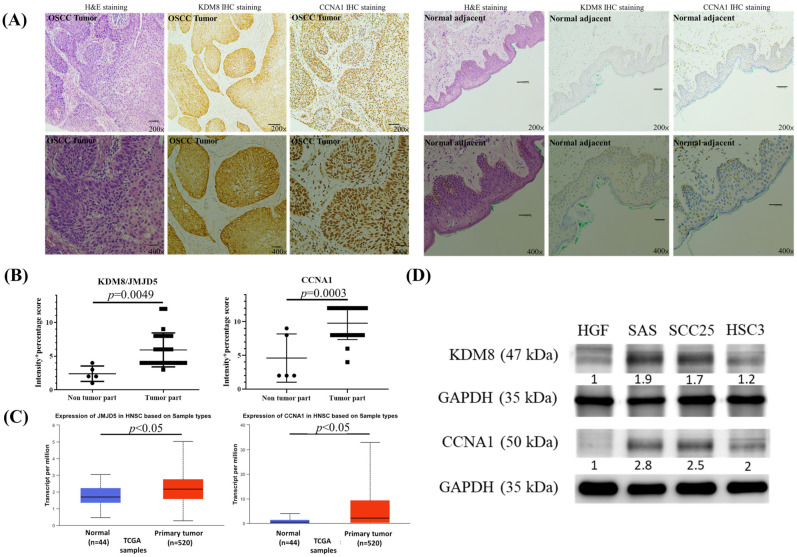
High expression of KDM8 existed in oral cancer. (**A**) H&E and KDM8 IHC stain for normal tissues adjacent to the tumor (magnification 200× and 400×). (**B**) Representative samples scored for KDM8 IHC results in normal mucosal and oral cancer tissues are shown. The score was defined as intensity score x percentage score. Data are expressed as mean ± standard deviation. *p* < 0.05 (Student’s *t*-test) represents a significant difference. (**C**) Expression levels of the KDM8 in HNSC patients based on the UALCAN database. (**D**) KDM8 and CCNA1 protein levels in oral cancer cell lines were determined through Western blot analysis. Normal human gingival fibroblast HGF-1 cells were used as the negative control.

At the beginning, we used immunohistochemical stain analysis to determine the expression status of the KDM8 and CCNA1 protein in 27 OSCC samples TMA (Table 1) and 5 normal oral mucosa samples adjacent to the tumor. Our findings, presented in Figure 1A,B, revealed a substantial increase in the levels of KDM8 and CCNA1 protein expression in the OSCC tumor samples relative to the normal mucosa samples. To validate our results, we utilized the UALCAN web tool, which utilizes the TCGA database, to analyze the mRNA levels of KDM8 and CCNA1 in HNSC tissues and normal tissues. Our analysis, presented in Figure 1C, showed a significant elevation in KDM8 and CCNA1 expression levels in HNSC tissues compared to normal tissues (*p* < 0.05). To further support our observations, we performed Western blotting analysis on various oral cancer cell lines (SAS, SCC25, and HSC3) and human normal gingival fibroblast cells (HGF) to investigate KDM8 and CCNA1 expression. Our results, illustrated in Figure 1D, demonstrated significantly higher levels of KDM8 and CCNA1 expression in the oral cancer cell lines relative to HGF cells.

### 3.2. AITC Exerts Cytotoxic Effects in Oral Cancer Cells

**Figure 2:**
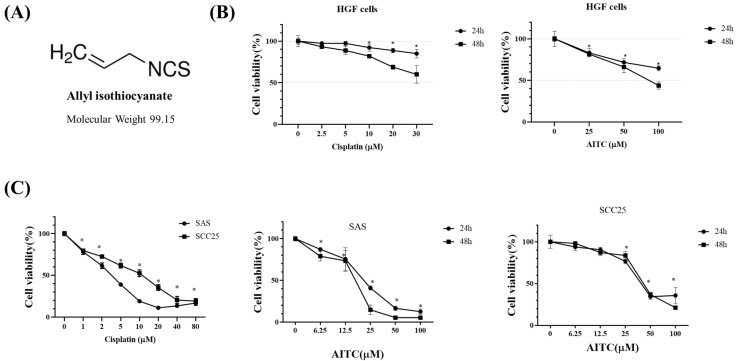
Effects of AITC on proliferation in SAS and SCC25 cells. The number of cells with methylene blue dye assay correspond to the mean ± SD of three independent experiments carried out in duplicate. (*) *p* < 0.05 compared to the negative control. (**A**) Structure of AITC. (**B**) Normal human gingival fibroblast HGF cells as the control. HGF cells were treated with various concentrations of cisplatin and AITC for 24 and 48 h, and then cell viability was determined by the methylene blue assay. (**C**) Cells were treated with various concentrations of cisplatin and AITC for 24 and 48 h, and then cell viability was determined by the methylene blue assay.

We investigated the impact of AITC (Figure 2A) on the cytotoxicity of normal human gingival fibroblast HGF (Figure 2B) and oral cancer cells (Figure 2C). The positive control chemotherapy cisplatin was also tested. To assess this, SAS and SCC25 oral cancer cells were exposed to varying concentrations of AITC for 24 and 48 h (as shown in Figure 2C). Our findings revealed that AITC exhibited a dose- and time-dependent inhibitory effect on the proliferation of tongue cancer cells (as depicted in Figure 2C). After 24 h of treatment, the IC_50_ of AITC for SAS was approximately 25 μM.

### 3.3. AITC Inhibited the Growth of Oral Cancer in Preclinical Models

**Figure 3:**
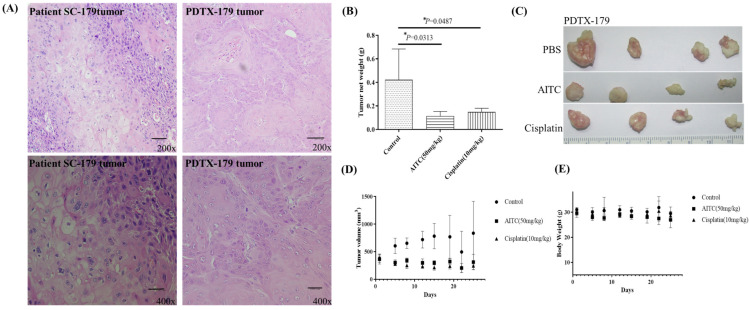
AITC inhibited tumor growth in oral cancer PDTX models. (**A**) H&E staining of the SC-179 clinical sample and PDTX-179. (**B**) In PDTX, NOD/SCID mice bearing patient-derived tumor tissue xenograft were treated with PBS (n = 4), AITC (n = 4; 50 mg/Kg/d), and cisplatin (n = 4; 10 mg/Kg/d). The average tumor weight of each group was compared with that of the control (*p* < 0.05 by Student’s *t*-test). (**C**) Tumor images for each group. (**D**) Changes in tumor volume in oral cancer PDTX models treated for 24 days. Diameters were measured twice a week for 24 days by using Vernier calipers, and the tumor volume was calculated as 1/2 × L × W^2^, where W and L are the shortest and longest diameters, respectively. Tumor volumes were compared with those of controls. All data are expressed as mean ± SD. *p* < 0.05 (Student *t*-test). (**E**) No significant change was observed in the mice’s body weight compared with that of the vehicle control. *: statistically significant difference.

**Figure 4:**
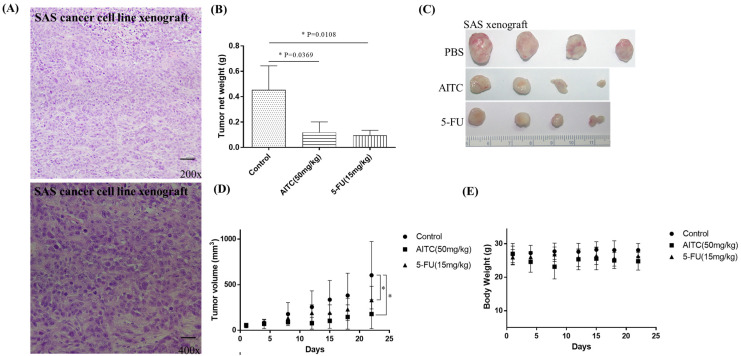
AITC inhibited oral cancer growth in SAS xenograft. (**A**) H&E staining of the SAS cancer cell linr xenograft. (**B**) In vivo, NOD/SCID mice bearing subcutaneous SAS cells were treated with PBS (n = 4), AITC (n = 4; 50 mg/Kg/d), and 5-FU (n = 4; 15 mg/Kg/d). The average tumor weight of each group was compared with that of the control (* *p* < 0.05 by Student’s *t*-test). (**C**) Tumor images for each group. (**D**) Changes in tumor volume in oral cancer SAS xenograft model, which was treated for 21 days. Diameters were measured twice a week for 21 days by using Vernier calipers, and the tumor volume was calculated as 1/2 × L × W^2^, where W and L are the shortest and longest diameters, respectively. Tumor volumes were compared with those of controls. All data are expressed as mean ± SD. * *p* < 0.05 (Student *t*-test). (**E**) No significant change was observed in the mice’s body weight compared with that of the vehicle control.

The initial chemotherapy approach for oral cancer can vary based on several factors, such as the cancer’s location and stage. Nonetheless, the most frequently employed chemotherapy method for oral cancer is a combination of cisplatin and 5-fluorouracil (5-FU). To determine the potential impact of allyl isothiocyanate (AITC) on PDTX proliferation in NOD SCID mice, we conducted further research. Our findings demonstrated that AITC, administered at a dosage of 50 mg/Kg, exhibited significant antitumor activity, similar to that of cisplatin administered at a dosage of 10 mg/Kg, compared to the PBS-treated control group (as depicted in Figure 3B). Additionally, the outcomes showed no discernible toxic effects or considerable loss of body weight among the different treatment groups (as illustrated in Figure 3C,D). Moreover, we assessed AITC’s in vivo cytotoxic potential by analyzing its capacity to suppress the growth of SAS xenograft oral cancer cells. The results revealed that AITC, administered at a dosage of 50 mg/Kg, demonstrated a substantial inhibition of tumor growth, which was akin to that of 5-FU (administered at a dosage of 15 mg/Kg) in the oral cancer xenograft (as demonstrated in Figure 4).

### 3.4. AITC Induced G2/M Cell Cycle Arrest and Apoptosis through the Modulation of G2/M-Associated Proteins KDM8 and CCNA1 in Oral Cancer Cells

**Figure 5:**
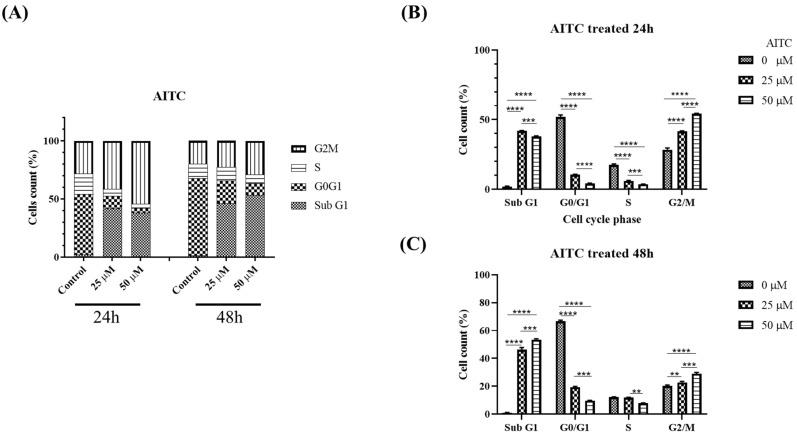
AITC induced oral cancer G2/M arrest. Flow cytometric cell cycle analysis of SAS cells treated with varying concentrations of AITC (25–50 μM) or DMSO (1 μL/mL) for 24 and 48 h. Graphs show cell cycle distribution (**A**) and distribution quantification percentage (**B**) for AITC treated 24 h and (**C**) AITC treated 48 h. **: statistically significant as *p* < 0.01; ***: statistically significant as *p* < 0.001; ****: statistically significant as *p* < 0.0001.

**Figure 6:**
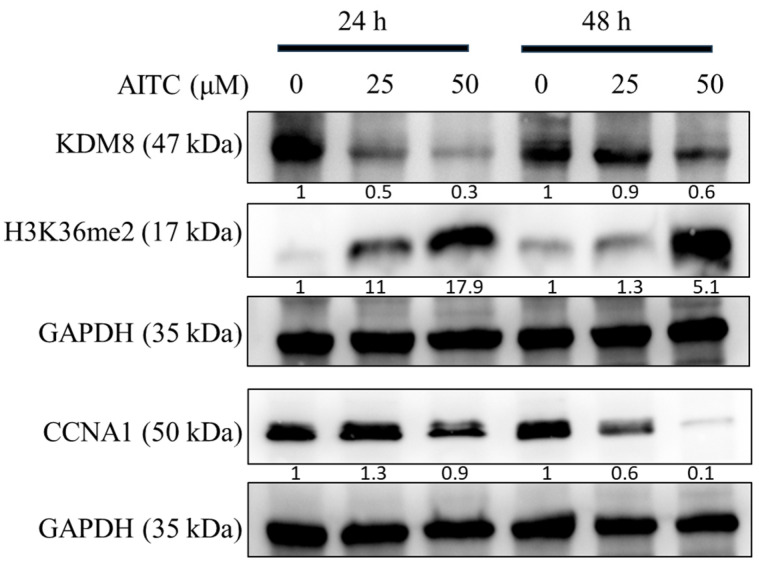
AITC downregulated KDM8 and CCNA1 expression in oral cancer cells. Western blot analysis and densitometry for KDM8, H3K36me2, and CCNA1 after SAS cells treated with AITC for 24 h and 48 h.

The AITC compound was found to induce cell cycle arrest at the G2/M phase, leading to reduced cell proliferation, potentially due to apoptosis, as evidenced by the sub-G1 population. This was determined through the examination of cell cycle progression profiles and the percentage of cells in each phase, as presented in Figure 5. A significant arrest at the G2/M phase and an increase in apoptotic cells in the sub-G1 groups were observed at a concentration of 25 μM of AITC. Additionally, AITC was observed to affect the levels of proteins linked to the G2/M phase. To confirm the buildup of the G2/M population in SAS oral cancer cells prompted by AITC, the levels of regulated proteins were evaluated. The results demonstrated a significant decline in KDM8 and CCNA1, and an increase in H3K36me2 in SAS cells exposed to AITC, as shown in Figure 6.

## 4. Discussion

KDM8, a member of an evolutionarily conserved protein family with a Jumonji domain, is currently recognized as an oncogene in colon and breast cancer [21,22,25,26]. In this study, we confirmed previous findings that KDM8 is overexpressed in OSCC [20,27]. KDM8 promotes breast cancer cell proliferation by activating CCNA1, a regulator of the G1/S and G2/M transition [13,54,55]. Additionally, KDM8 inhibits p21, a potent inhibitor of a cyclin-dependent kinase, which leads to cell growth retardation, the loss of pluripotency in embryonic stem cells [56], and embryonic lethality when KDM8 is deficient in mouse embryonic cells [57]. These results provide strong evidence that KDM8 is crucial in regulating the G2/M cell cycle. Furthermore, knocking down KDM8 using siRNA inhibited the growth of oral cancer cells (Figure S2). The results showed a similarity with a previous study [20]. Previously conducted studies have indicated that allyl isothiocyanate (AITC) can prompt G2/M cell cycle arrest in various types of cancer, such as brain glioma, colorectal adenocarcinoma, and breast adenocarcinoma[40,58,59]. CCNA1, which is a member of the highly conserved cyclin family, may be involved in regulating the cell cycle at both G1/S and G2/M transition points [6]. Based on immunohistochemistry-based tests, several studies have suggested that CCNA1 may be linked to poor prognosis in OSCC [12,60]. In the current study, we discovered that AITC could cause G2/M arrest in OSCC cells and reduce CCNA1 expression. Thus, these results imply that the potential anti-oral cancer effects of AITC may be associated with the suppression of CCNA1.

Histone modification is a crucial mechanism of epigenetic regulation. During the post-translational modification (PTM) of histones, histone methylation marks have various effects on transcriptional activation [61]. Several studies have shown that the global regulation of the H3K36me2 histone mark is responsible for epithelial plasticity and metastatic progression [62]. Furthermore, Jumonji domain 2 (JMJD2/KDM4) cluster members are capable of demethylating H3K36me3/2 [61,63]. These JMJD2 family members are overexpressed in various human cancers [7,28,29], indicating that H3K36me3 may serve as a tumor suppressor marker. In this study, our findings suggest that the downregulation of KDM8 expression by AITC treatment could induce H3K36me2 expression.

Although AITC has shown promise in its ability to combat cancer, there are still obstacles to its clinical application [29,64]. PDTX is a type of preclinical cancer model where the tumor tissue from a patient is implanted into immunodeficient mice to create a tumor that closely retains the tumor heterogeneity present in the original patient sample, which enables them to replicate the complexity of the tumor, including the tumor microenvironment. In this study, we created a PDTX model which is actually similar to the original tumor by histopathological analysis (H&E stain) (Figure S1). This feature is critical for drug development [65]. PDTX models have several advantages for cancer research and drug development, including having good biological relevance, being more predictive of patient response to treatment than cell line models, being suitable for personalized medicine, and having high fidelity. PDTX models can be propagated indefinitely, allowing for long-term studies and drug development. Additionally, PDX models can be used to study tumor evolution and drug resistance, which is difficult to do in patients due to ethical and practical limitations. However, there are also many disadvantages for PDTX models which are costly and time-consuming, have limited engraftment success, and show variability in tumor growth rates. PDTX models may exhibit variations in tumor growth rates, which can complicate drug testing standardization. In this study, our results in Figure 3 have shown variations in tumor growth volume and tumor mass. Although different cancer types of preclinical PDTX models have been established, research on PDTX models for oral cancer is limited [66]. To address this gap, this study aimed to establish PDTX models and verify the anticancer activity of AITC in oral cancer.

## 5. Conclusions

AITC inhibited oral cancer growth in vitro, in vivo, and in PDTX models. KDM8 and CCNA1 repression by AITC markedly suppressed oral cancer’s proliferation or growth. Thus, AITC might be the candidate adjuvant of OSCC. However, further study should still reveal the mechanism of the AITC-regulated anticancer effect.

## Figures and Tables

**Table 1:** The clinical parameters of OSCC patients in TMA (*n* = 27).

**Clinical parameters of the OSCC patients included in this study**	
**Characteristics**	Patients (*n* = 27)
**Gender**	
Male	23
Female	4
**Age**	
Range	37–82
Median	59.5
**Tumor Size**	
T1–T3	18
T4	9
**Clinical Stage**	
Ⅰ–III	15
Ⅳ	12
**Lymph node**	
LN (−)	13
LN (+)	14
**Location**	
Lip	2
Anterior pilla	1
Gingiva	5
Tongue	10
Soft palate	1
Buccal mucosa	7
Mouth floor	1
**Recurrent**	4
**Death**	3

## Data Availability

All data created or analyzed during this study are available from the corresponding author upon reasonable request.

## Supplementary Materials

The following supporting information can be downloaded at: https://www.mdpi.com/article/10.3390/biomedicines11102669/s1, Figure S1: The PDTX establishment process. Figure S2: KDM8 suppression alleviates OSCC proliferation.
