# Supplementary material for: Allyl Isothiocyanate Suppresses the Proliferation in Oral Squamous Cell Carcinoma via Mediating the KDM8/CCNA1 Axis
Source: Biomedicines. 2023 Sep 29;11(10):2669. doi: 10.3390/biomedicines11102669 (PMC10604360; doi:10.3390/biomedicines11102669)
Supplement: Supplementary file 1 [file biomedicines-11-02669-s001.zip › biomedicines-2563174-supplementary.pdf]

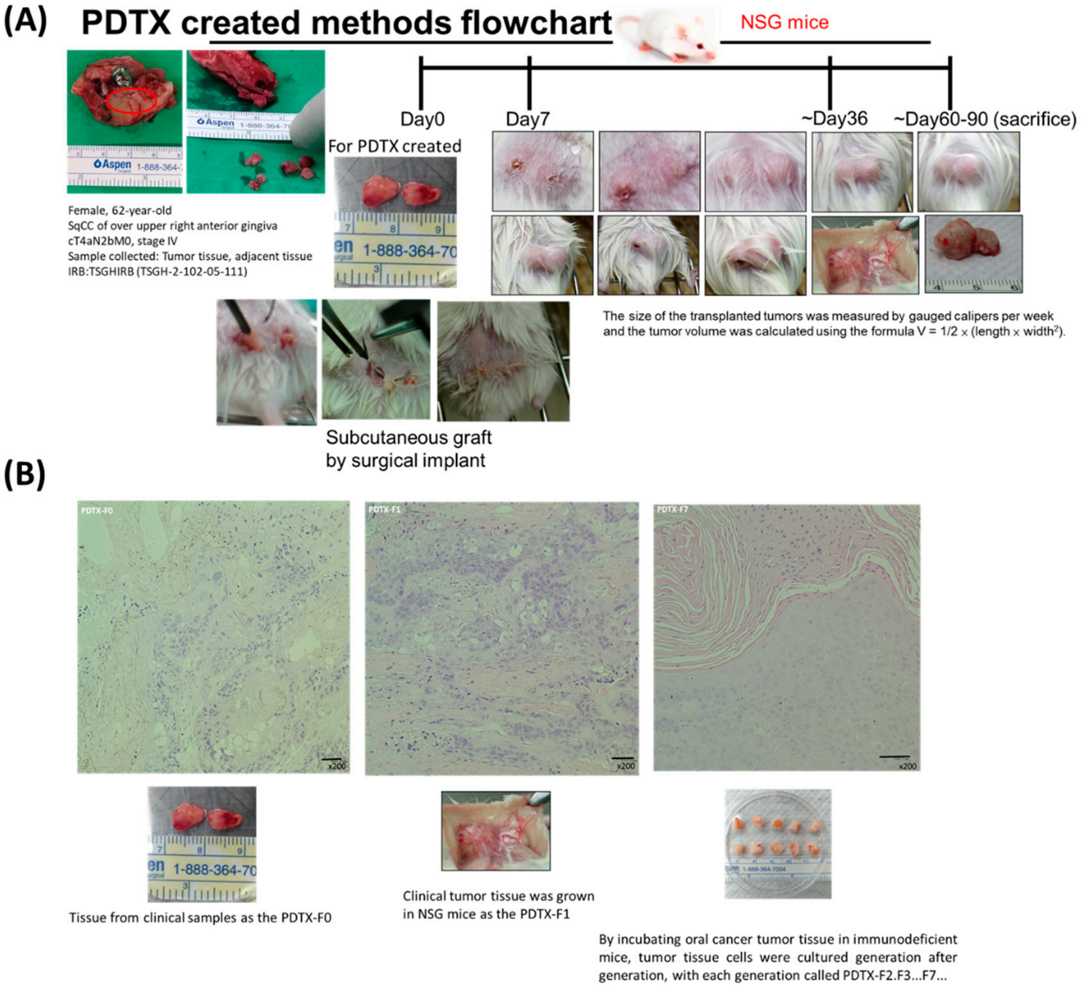

Figure S1. (A) presents a flowchart outlining the PDTX establishment process. (B) Histopathological analysis (H&E stain). PDTX-F0 represents the oral cancer tumor tissue obtained from clinical specimens for PDTX model creation. Clinical tumor tissue was grown in NSG mice as the PDTX-F1. By incubating oral cancer tumor tissue in immunodeficient mice, tumor tissue cells were cultured generation after generation, with each generation called PDTX-F2. F3...F7.

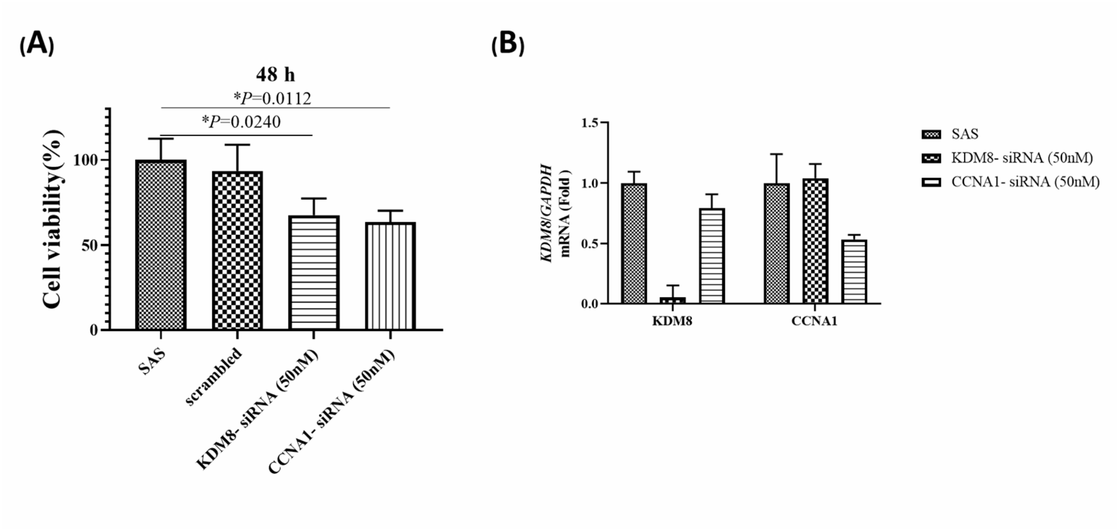

Figure S2. KDM8 suppression alleviates OSCC proliferation. (A) SAS cancer cells were transfected with KDM8 siRNA and CCNA1 siRNA sequences for 48 h, followed by RT-qPCR of KDM8 and CCNA1. The siRNA sequences were obtained from Dharmacon (M-003983-02-0005, M-003204-02-0005). The target sequence for KDM8: CCACUGAGCUCUUCUACGA, GAAGUUGGUUCGAGGUACA, UCAACGAGUUCAUCAGCAA, and AGCCAAGGGACGUCGGGUA. Target sequence for CCNA1: GAACCUGGCUAAGUACGUA, GCAGAUCCAUUCUUGAAAU, UCACAAGAAUCAGGUGUUA, and CAUAAAGCGUACCUUGAUA.
